# Potent social synchronization can override photic entrainment of circadian rhythms

**DOI:** 10.1038/ncomms11662

**Published:** 2016-05-23

**Authors:** Taro Fuchikawa, Ada Eban-Rothschild, Moshe Nagari, Yair Shemesh, Guy Bloch

**Affiliations:** 1 Department of Ecology, Evolution and Behavior, The Alexander Silberman Institute of Life Sciences, The Hebrew University of Jerusalem, Givat-Ram, Jerusalem 91904 Israel

## Abstract

Circadian rhythms in behaviour and physiology are important for animal health and survival. Studies with individually isolated animals in the laboratory have consistently emphasized the dominant role of light for the entrainment of circadian rhythms to relevant environmental cycles. Although in nature interactions with conspecifics are functionally significant, social signals are typically not considered important time-givers for the animal circadian clock. Our results challenge this view. By studying honeybees in an ecologically relevant context and using a massive data set, we demonstrate that social entrainment can be potent, may act without direct contact with other individuals and does not rely on gating the exposure to light. We show for the first time that social time cues stably entrain the clock, even in animals experiencing conflicting photic and social environmental cycles. These findings add to the growing appreciation for the importance of studying circadian rhythms in ecologically relevant contexts.

## Abstract (web summary)

Circadian rhythms synchronize important biological processes, and are thought to primarily be entrained by environmental cycles in light and temperature, with little or no role for social interactions. Here, Fuchikawa *et al*. show that social cues among honeybees can entrain these rhythms even in the presence of conflicting light-dark cycles.

## Graphical abstract

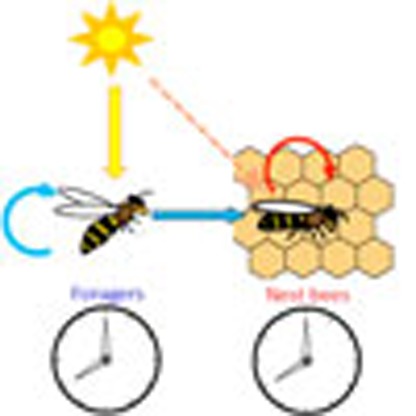

Circadian clocks driving rhythms in behaviour and physiology are ubiquitous in metazoans, including animals living in constant environments^1^,^2^,^3^. The fitness benefit of these internal clocks is thought to lay in the temporal organization and coordination of internal molecular, biochemical and physiological processes^4^,^5^,^6^, and in aligning these processes with regular and predictable day–night cycles in the environment^7^,^8^. Light and temperature are considered the dominant time-givers (‘zeitgebers') entraining circadian clocks in diverse organisms including cyanobacteria, fungi, plants and animals^9^. Progress in molecular genetics and the development of methods allowing high resolution and precise monitoring of circadian rhythms in locomotor activity for isolated individuals in a constant laboratory environment has enabled remarkable progress in deciphering the mechanisms underlying the generation of circadian rhythms and their photic entrainment (for example, mammals^10^ and flies^11^). However, in the wild, animals live in complex environments in which their ability to adjust their behaviour to respond to conspecifics has profound fitness consequences^12^,^13^. Even in modern human societies in which individuals have unprecedented control on their environment, there is evidence that the discrepancy between biological (circadian) and societal timing (such as school or work schedules) results in ‘social jetlag' that can be expressed in reduced performance and increased illness^14^,^15^,^16^. Thus, it is reasonable to assume that social influences on the circadian clock may be important in many, if not all animals, but currently there is only little support for this premise.

If social entrainment is indeed important, then there should be pathways connecting the perception of social signals to the endogenous clock system. To this end, however, no neuronal pathways transmitting visual, auditory, olfactory or tactile social stimuli to the clock have been described. Rather, it has been suggested that social influences on the mammalian clock are mediated by neuronal and/or endocrine correlates of nonspecific arousal or increased activity^17^, by sleep^18^, or by learning processes that associate circadian time with specific actions^19^. Given that the light–dark (LD) cycle is the dominant environmental factor influencing circadian rhythms, it was further proposed that social stimuli can affect circadian functions by modulating the time and pattern of exposure to potent photic time-givers^17^. This notion has also been applied to humans, in which the early view that social cues have a necessary and sufficient role as circadian time-givers independent of light has reversed and light is now viewed as the primary, if not exclusive, circadian time-giver^17^,^20^.

An additional difficulty with social entrainment is the lack of a clear conceptual framework. It is sensible to assume that social entrainment is more important for group living than for solitary animals, but this hypothesis has received little support. There is indeed profound species-specific variation in studies testing social entrainment, but there is no simple relationship between the degree of sociality and the sensitivity of the circadian system to social influences^21^. Although some of this variability can be attributed to differences in experimental design, it is notable that social interactions failed to entrain circadian rhythms in social mammals such as the squirrel monkey *(Saimiri sciureus*), Mongolian gerbil (*Meriones unguiculatus*) and sugar gliders (*Petaurus breviceps*)^22^,^23^,^24^, as well as in the gregarious cockroach *Leucophaea maderae*^25^. On the other hand, convincing social influences on circadian rhythms have been found in species that are not considered social, such as the fruit fly^26^. Even time-specific interactions with a sexual partner, which are expected to have clear fitness benefits, have produced variable results, including studies in which they failed to have even small influences on circadian phase^27^,^28^. The best evidence for social entrainment is found in dark cavity-dwelling social animals such as bees and bats. These species may be especially responsive to social entrainment, because individuals may not experience ambient conditions directly, but rather rely on information received from group mates that forage outside their domicile^12^,^29^.

We studied the highly social honeybee *Apis mellifera* as a model for testing the hypothesis that social cues provide robust entraining signals, even capable of overriding strong photic signals. Social insects are ecologically important and offer attractive model systems for studies on the interplay between social behaviour and circadian rhythms. The temporal coordination of their activities is thought to be imperative for efficient colony functioning and for colony fitness (reviewed in refs 21, 30). During their pre-adult development and the first 2–3 weeks as adults, honeybee workers typically reside inside the constantly dark and tightly thermoregulated cavity of the nest. Young adult workers typically tend (‘nurse') the developing eggs, larvae and pupae (collectively termed ‘brood') around the clock with no circadian rhythms in activity and in whole-brain clock-gene mRNA levels^31^,^32^,^33^,^34^. Nevertheless, these around-the-clock active bees do have a functional and entrained circadian clock; nurse bees that are removed from the hive and isolated individually in a constant laboratory environment almost instantly show circadian rhythms with high locomotor activity during the subjective day and relative quiescence during the subjective night^31^,^34^.

We used field-foraging honeybee colonies, allowing us to study social entrainment in an ecologically relevant context. We first show that nest bees as young as 2 days of age are strongly entrained by the colony phase. We then show that this potent social entrainment does not depend on physical contact with other bees in the colony. Finally, we show that young nest bees that experience conflicting social and LD cycles maintain the phase of the social cycle when removed from the hive and monitored individually in constant laboratory conditions. This study demonstrates a remarkably potent social entrainment and, to the best of our knowledge, provides the first evidence that social entrainment can override photic entrainment.

## Results

### Young nest bees are entrained to ambient day–night cycles

**Figure 1:**
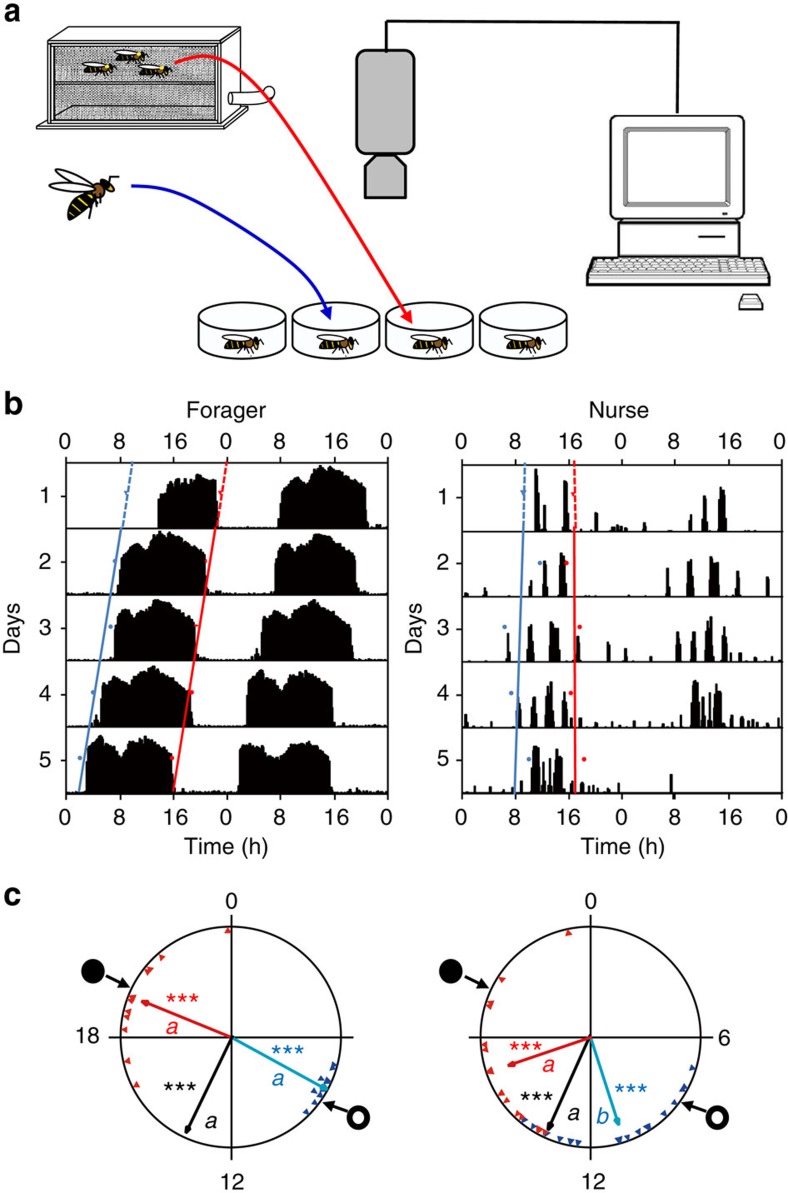
Nurses removed from field-foraging colonies are synchronized to the day–night cycle outside. (**a**) Experimental procedure. Nurses and foragers were collected from a field-foraging observation hive. The hive was constantly dark but foragers exited the hive and are assumed to have experienced 12 h light (natural light)/12 h dark (LD) cycles. On the eighth day, we collected foragers and nurses, placed each individually in a cage and monitored their locomotor activity for 5 days in constant conditions. (**b**) Representative double-plot actograms. Each panel shows locomotor activity for an individual bee monitored in constant laboratory environment. The *y* axis depicts days in the monitoring cage; the height of the bars within each day corresponds to the level of locomotor activity in a 10-min bin. The blue and red dots show the time of onset and offset of activity for each day, respectively. Regression lines with corresponding colours are fitted to these points. The time of onset and offset on the day on which the bee was transferred from the hive to the laboratory (day 1) was extrapolated from the regression models. (**c**) Representative circular statistics for sister nurses and foragers from colony H7. The time of day is depicted on the circular plot perimeter. The open and filled circles on the perimeter delineate the time of sunrise and sunset, respectively. The blue and red triangles depict the onset and offset for individual bees, respectively. The blue, black and red vectors (arrows) pointing from the centre towards the perimeter show the average time for the onset, median and offset, respectively. Vector length corresponds to the extent of phase coherence. Asterisks in matching colours correspond to the *P*-value obtained from a Rayleigh test for phase coherence (*0.01<*P*<0.05, **0.001<*P*<0.01 and ***P<0.001). The median for each bee (points for individual bees are not shown) was calculated as the midpoint between the onset and offset. Vectors sharing the same colour across different plots, and which are marked with different small letters, are significantly different in a Watson–Williams F-test across experimental groups. Sample size is 11 and 17 for foragers and nurses, respectively.

Do young nest bees that are typically active around-the-clock in a constantly dark hive nevertheless have clocks that are entrained with the ambient day–night cycle? In the first experiment we found that nurses in a dark hive show strong circadian rhythms in locomotor activity shortly after transfer to the laboratory in both trials (with bees from colonies H7 and S8). They showed a coherent phase when using the onset, median or offset as an index for phase (Rayleigh test, length of vector: 0.939–0.673; colony H7, *n*=17, 15 and 15; colony S8, *n*=18, 17 and 17, respectively, *P*<0.001; Fig. 1, Supplementary Fig. 1 and Supplementary Table 1). The onset of their daily bout of activity tended to be delayed relative to that of foragers (statistically significant only in colony H7; Watson–Williams F-test, *n*=11 and 17 for ‘Forager' and ‘Nurse', respectively, *P*<0.001; colony S8, *n*=31, 13 and 18, *P*=0.22). The offset of activity was earlier in nurses in the trial with bees from colony H7. Similar differences between the onset and offset were also found in previous studies in which the nurses were collected from LD-illuminated hives^31^,^34^. The median between the onset and offset was similar for foragers and nurses in both trials (Watson–Williams F-test, colony H7, *n*=11 and 15 for ‘Forager' and ‘Nurse', respectively, *P*=0.86; colony S8, *n*=13 and 17, *P*=0.62; Fig. 1 and Supplementary Fig. 1). These results indicate that although nurses are typically active around the clock in the dark and homeostatically regulated nest, they nevertheless have functional pacemakers that are synchronized with ambient day–night cycles.

**Figure 2:**
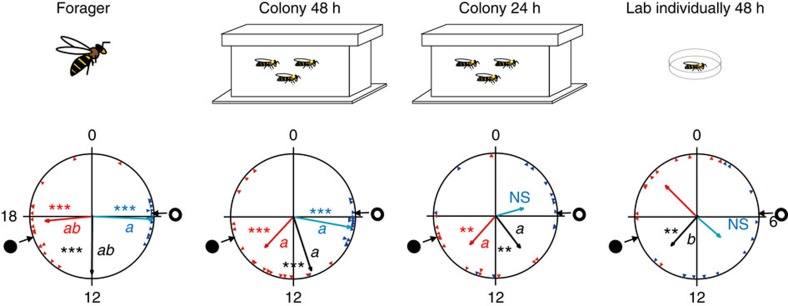
Forty-eight hours in the hive are sufficient for strong synchronization of newly emerged bees to the colony phase. Representative circular statistics for foragers and callow bees from colony H11. ‘Colony 48 h'—young bees that experienced their first 48 h in a field colony and then monitored individually in constant laboratory conditions. ‘Colony 24 h'—same as above, but bees experienced the colony environment for only 24 h. ‘Lab individually 48 h'—young bees that experienced their first 48 h isolated individually and then monitored individually in constant laboratory conditions. The open and filled circles on the perimeter delineate the time of sunrise and sunset, respectively. The blue and red triangles depict the onset and offset for individual bees, respectively. The blue, black and red vectors (arrows) pointing from the centre towards the perimeter show the average time for the onset, median and offset, respectively. Vector length corresponds to the extent of phase coherence. Asterisks in matching colours correspond to the *P*-value obtained from a Rayleigh test for phase coherence (*0.01<*P*<0.05, **0.001<*P*<0.01 and ***P<0.001). The median for each bee (points for individual bees are not shown) was calculated as the midpoint between the onset and offset. Vectors sharing the same colour across different plots, and which are marked with different small letters, are significantly different in a Watson-Williams F-test with Bonferroni *post-hoc* tests across experimental groups. Sample sizes are 15, 17, 13 and 11 for ‘Forager', ‘Colony 48 h', ‘Colony 24 h' and ‘Lab individually 48 h', respectively. The cartoons at the top summarize the social conditions experienced by the bees before transferring to locomotor activity monitoring cages in the laboratory.

We next asked whether the hive environment can similarly synchronize circadian rhythms at a younger age, at which bees typically do not show circadian rhythms even when placed in individual cages outside the hive. We addressed this question in Experiment 2 in which we determined circadian rhythms and synchronization for newly emerged bees that were placed in individual cages shortly after emerging from the pupae or after development for 1 or 2 days in the hive. Our analyses suggest that development for 2 days in the colony, but not one, is sufficent for achieving good synchronization with the hive environment (Fig. 2, Supplementary Fig. 2 and Supplementary Tables 2 and 3). The bees that developed for 2 days in the colony (‘Colony 48 h') showed strong circadian rhythms, with high levels of activity during the subjective day and with a coherent phase, similar to the findings for older nurses in Experiment 1. As normal-age nurses, ‘Colony 48 h' bees also tended to have a later onset and earlier offset relative to the foragers, but these trends did not reach statistical significance (Watson–Williams F-test with Bonferroni *post-h*oc tests, onset: *n*=15 and 17 for ‘Forager' and ‘Colony 48 h', respectively, *P*=0.21; offset: *n*=15 and 16, respectively, *P*=0.025). Sister bees of a similar age that were individually isolated in the laboratory for 48 h (‘Lab individually 48 h') showed a different pattern. They typically had weaker rhythms, reduced phase coherence, which, in the repetitions where their phase coherence was statistically significant, tended to differ from that of their sisters in the hive (colony H11 median and offset; colony S73, onset; Fig. 2 and Supplementary Fig. 2). Young bees that experienced the colony environment for only 24 h tended to have a weaker phase coherence compared with their sister bees that experienced the colony environment for 48 h and in many cases their phase coherence was not statistically significant (Fig. 2, Supplementary Fig. 2 and Supplementary Tables 2 and 3). Foragers showed strong circadian rhythms with elevated activity during the subjective day and strong phase coherence as in Experiment 1 and previous studies^31^,^34^. These results show that even at the age of 2 days, most bees have a functional clock that can be entrained by time-givers in the hive.

**Figure 3:**
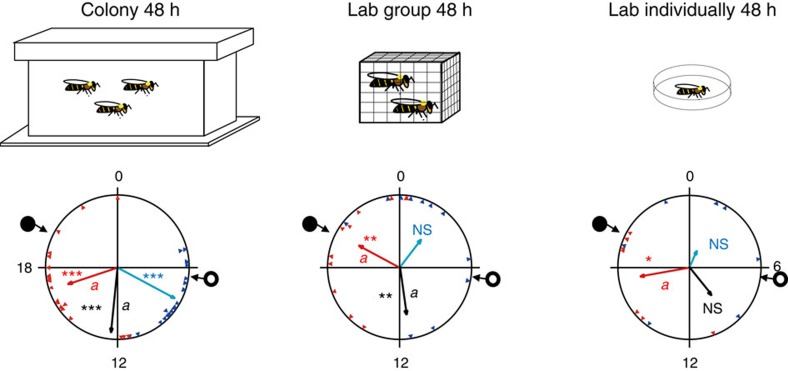
Social interactions with 30 bees produce weaker synchronization than the hive environment. Representative circular statistics for callow bees from colony H6. ‘Lab group 48 h'—young bees that experienced their first 48 h in a cage with 30 other same-age bees before being monitored individually in constant laboratory conditions. The open and filled circles on the perimeter delineate the time of sunrise and sunset, respectively. The blue and red triangles depict the onset and offset for individual bees, respectively. The blue, black and red vectors (arrows) pointing from the centre towards the perimeter show the average time for the onset, median and offset, respectively. Vector length corresponds to the extent of phase coherence. Asterisks in matching colours correspond to the *P*-value obtained from a Rayleigh test for phase coherence (*0.01<*P*<0.05, **0.001<*P*<0.01 and ***P<0.001). The median for each bee (points for individual bees are not shown) was calculated as the midpoint between the onset and offset. Vectors sharing the same colour across different plots, and which are marked with different small letters, are significantly different in a Watson-Williams F-test with Bonferroni *post-hoc* tests across experimental groups. Sample sizes are 21, 11 and 7 for ‘Colony 48 h', ‘Lab group 48 h' and ‘Lab individually 48 h', respectively. The cartoons at the top summarize the social conditions experienced by the bees before transferring to locomotor activity monitoring cages in the laboratory.

The improved synchronization in young bees that experienced the hive environment compared with their individually isolated sisters can be explained by direct social interactions with nestmates or by a more diffused synchronizing effect of the hive environment. To distinguish between these explanations we compared (Experiment 3) the synchronization of young bees that were placed in individual cages, in groups of 30 bees outside the hive, or free to move within the hive (Fig. 3). The bees in the cage outside the hive (‘Lab group 48 h') could socially interact with each other, but did not experience the colony environment. The phase coherence in these small groups was intermediate between the strong synchronization in the colony (‘Colony 48 h') and the weak synchronization of the individually isolated bees (‘Lab individually 48 h') (mean length of vector, onset: Colony 48 h, Lab group 48 h and Lab individually 48 h=0.910, 0.689 and 0.327, respectively; median: 0.889, 0.764 and 0.546, respectively; offset: 0.634, 0.620 and 0.644, respectively; Fig. 3, Supplementary Fig. 3 and Supplementary Table 4 and 5). The median in colony H12 and onset in colony H11 differed between the laboratory caged bees and their same-age sisters that experienced the hive environment for 48 h (Watson-Williams F-test with Bonferroni *post-hoc* tests, median in colony H12: *n*=22, 9, and 6, for ‘Colony 48 h', ‘Lab group 48 h' and ‘Lab individually 48 h', respectively, *P*<0.02 for the comparison between ‘Colony 48 h' and ‘Lab group 48 h'; onset in colony H11: *n*=16, 7, and 10, *P*<0.001 for the comparison between ‘Colony 48 h' and ‘Lab group 48 h'). These findings suggest that social interactions within a group of 30 bees of the same age enclosed in a cage can lead to phase synchronization, but this social entrainment is weak compared with the synchronizing effect of the whole colony environment.

### Physical contact is not necessary for social synchronization

**Figure 4:**
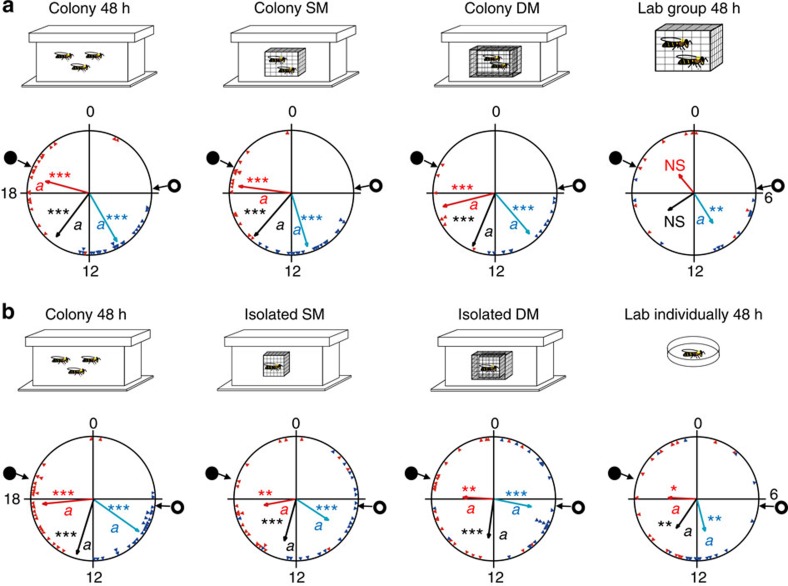
Direct contact with other bees is not needed for entrainment by the colony environment. (**a**) Representative circular statistics for callow bees from colony H2. ‘Colony SM'—young bees that were caged in a SM enclosure together with 30 sister bees inside a field-foraging colony before being monitored individually in constant laboratory conditions. ‘Colony DM'—same as Colony SM but the enclosure had two meshes that were 1.5 cm apart. The open and filled circles on the perimeter delineate the time of sunrise and sunset, respectively. The blue and red triangles depict the onset and offset for individual bees, respectively. The blue, black and red vectors (arrow) pointing from the centre towards the perimeter show the average time for the onset, median and offset, respectively. Vector length corresponds to the extent of phase coherence. Asterisks in matching colours correspond to the *P*-value obtained from a Rayleigh test for phase coherence (*0.01<*P*<0.05, **0.001<*P*<0.01 and ***P<0.001). The median for each bee (points for individual bees are not shown) was calculated as the midpoint between the onset and offset. Vectors sharing the same colour across different plots, and which are marked with different small letters, are significantly different in a Watson-Williams F-test with Bonferroni *post*-*hoc* tests across experimental groups. Sample sizes are 28, 24, 18 and 14 for ‘Colony 48 h', ‘Colony SM', ‘Colony DM' and ‘Lab group 48 h', respectively. (**b**) Same as in **a** but each newly emerged bee was caged individually in a SM or DM enclosure in the colony. Repetition with bees from colony H11. Sample sizes are 25, 22, 24 and 16 for ‘Colony 48 h', ‘Isolated SM', ‘Isolated DM' and ‘Lab individually 48 h', respectively.

We used single- (SM) and double-mesh (DM) enclosures to manipulate interactions between sister bees within a colony (Experiment 4). Young bees tested after group confinement into a SM or a DM enclosure showed strong rhythms (Fig. 4a and Supplementary Figs 6 and 7) and a coherent phase that was synchronized with the ambient day–night cycles (Rayleigh test, *n*=24, 17 and 21 for ‘Colony SM'; *n*=18, 7 and 13 for ‘Colony DM'; for onset, median and offset, respectively; *P*<0.001; Fig. 4a, Supplementary Fig. 4a and Supplementary Table 6). In all three trials, their phase coherence did not differ from that of sister bees freely moving within the hive (‘Colony 48 h'), but was typically stronger than that of caged bees in groups of 30 (‘Lab group 48 h') (Fig. 4a, Supplementary Fig. 4a and Supplementary Table 7). As interactions inside the mesh enclosures may have synchronized the bees in Experiment 4, we performed a complementary experiment (Experiment 5) in which we caged bees individually in SM or DM enclosures inside field colonies. Bees that were caged individually in SM or DM enclosures were synchronized with each other and with the day–night cycles, similar to the bees caged in groups in Experiment 4 (Fig. 4b, Supplementary Fig. 4b and Supplementary Table 8). Their phase (onset, median and offset) did not differ from that of their sisters freely moving within the hive. However, their phase coherence appeared weaker than that of freely moving bees (Fig. 4b, Supplementary Fig. 4b and Supplementary Table 9). The bees that were individually isolated outside the hive also showed evidence of synchronization. In the repetition with colony H11, their phase coherence was weak (Fig. 4b) but their average phase did not differ from the other groups; in the repetition with colony H2, only a few of these bees showed significant circadian rhythms, but their phase was coherent (Supplementary Fig. 4b). However, their average phase differed from that of bees moving freely in the hive for all three indices. We assume that the relatively strong synchronization of the individually isolated bees in this experiment compared with the other experiments was because their cages were placed close to each other and transferred cues such as vibrations or volatile odours. Taken together, these experiments indicate that physical contact with other bees in the hive is not needed for synchronization by the hive environment. Given that the mesh enclosures confined the bees to the inner part of the hive, these results further indicate that exposure to light at the hive entrance, or visits to other weakly regulated peripheral parts of the hive cannot account for the strong synchronization of the young bees.

### Potent social time-givers override photic entrainment

**Figure 5:**
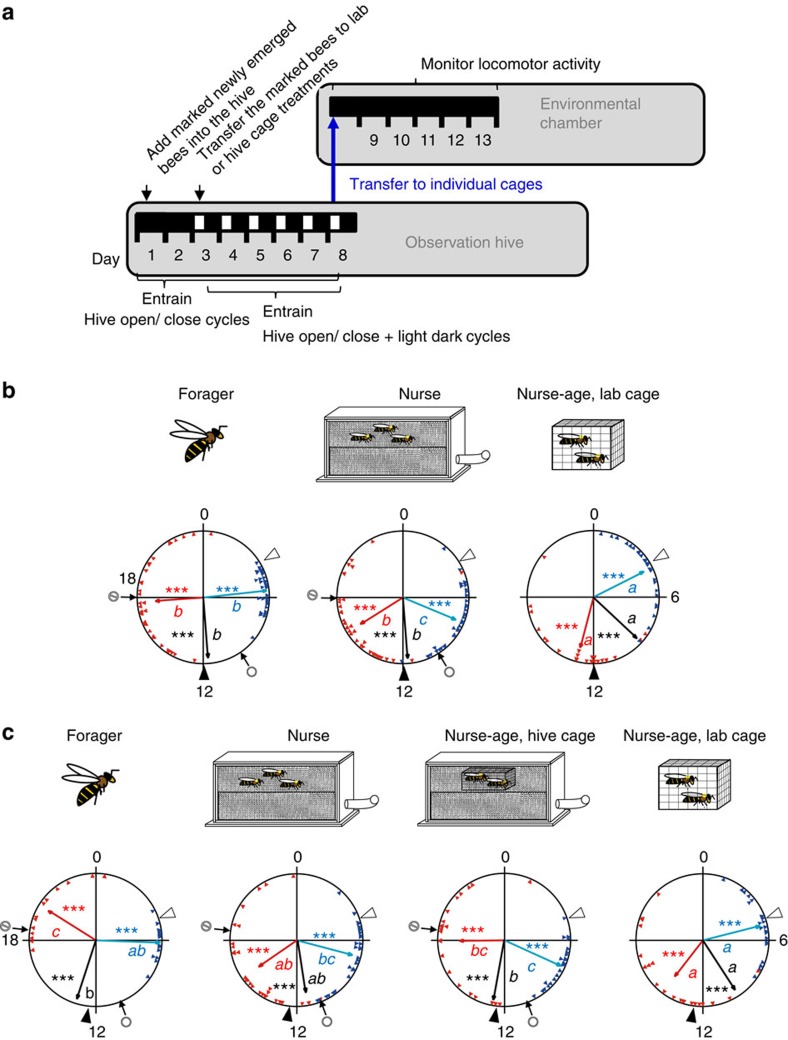
The colony cycle socially entrains bees experiencing conflicting photic and social time-givers. (**a**) Experimental outline. The lower horizontal bar shows the days after introducing newly emerged bees into the observation hive and the illumination regime in the observation hive room: open box, light; filled box, darkness. Starting on day 1, the entrance to the hive was opened only at limited times during the day. During days 3–8, the bees experienced conflicting hive opening/closing and light dark cycles. When the bees were 8 days of age, they were transferred (blue arrow) to individual monitoring cages in constant laboratory conditions and their locomotor activity was monitored for 5 days. (**b**) Representative circular statistics for bees from colony O12. The nurses and foragers were collected from an observation hive with conflicting light–dark and foraging activity cycles. ‘Nurse-age, lab cage'—a group of 30 newly emerged bees were housed in a cage that was placed next to the observation hive and experienced only the light–dark cycles. The bees from all groups were transferred to locomotor activity monitoring cages at the age of 8 days. Sample sizes are 34, 35 and 24 for ‘Forager', ‘Nurse' and ‘Nurse-age, lab cage', respectively. (**c**) Representative circular statistics for bees from trial 4 (colony 13–13). The first three treatments are the same as in **b**. This trial had an additional treatment that consisted of newly emerged bees that were caged in a SM enclosure inside the hive (‘Nurse-age, hive cage'). Sample sizes are 15, 28, 22 and 21 for ‘Forager', ‘Nurse', ‘Nurse-age, hive cage' and ‘Nurse-age, lab cage', respectively. The circle and the circle-backslash symbols point to the times of opening and closing the hive entrance, respectively. Open and filled arrowheads point to the times of lights-on and -off in the chamber in which the observation hive and cage were housed. Asterisks in matching colours correspond to the *P*-value obtained from a Rayleigh test for phase coherence (***P<0.001). Vectors sharing the same colour across different plots, and which are marked with different small letters, are significantly different in a Watson-Williams F-test with Bonferroni *post-hoc* tests across experimental groups.

Given that the experiments above showed that the colony environment effectively entrains circadian rhythms in young honeybees, we next examined whether this social time-giver can compete with strong photic entrainment. To address this question we studied entrainment for young bees experiencing conflicting colony environment and LD illumination cycles (Experiment 6). Cycles in the colony environment were manipulated by controlling the opening and closing of the hive entrance, which determined the time of foraging activity. Control nurse-age bees that were caged outside the hive and experienced only the LD illumination cycles (‘Nurse-age, lab cage') showed strong entrainment, with their onset and offset of activity strongly correlated with the time of lights-on and -off, respectively (Fig. 5b,c and Supplementary Fig. 5; the phase was somewhat delayed in the third trial with colony 13–10; Supplementary Fig. 5b). The robust synchronization of this control group indicates that young bees are effectively entrained by the illumination cycle used in our experiments. The foragers and the nurses showed strong phase coherence; as in the other experiments, the onset was earlier and the offset later in foragers relative to that of nurses (Fig. 5b,c and Supplementary Fig. 5). In all four trials the offset of forager activity was at around the time of closing the hive entrance and clearly later than the time of lights off, and the offset of activity for the nurse-age bees caged outside the hive. Although the onset of forager activity was advanced relative to the time of hive opening, it was still delayed relative to that of nurse-age bees in the laboratory in the first two trials (Fig. 5b and Supplementary Fig. 5a). A similar trend was also seen in the other two trials (Fig. 5c and Supplementary Fig. 5b). The delayed activity phase in foragers relative to the nurse-age bees in the laboratory was also apparent in the analyses of the median, which was significantly delayed in the foragers in three of the four trials (a similar trend was also seen in the third trial with colony 13–10; Supplementary Fig. 5b). Both the offset and median of nurse activity were more similar to that of foragers than to their same-age sisters that were caged in the laboratory in three of four trials. Taken together, these results suggest that the most important factor setting the phase of forager activity was the opening and closing of the hive entrance, with relatively little influence to the time of lights-on, and apparently no effect to the time of lights-off, inside the hive. The activity phase of the nurses in the hive was more similar to the foragers than to the nurse-age bees in the laboratory, indicating that the forager activity has a stronger influence on their phase than the LD cycles.

In the last two trials, we restricted an additional group of newly emerged bees to the inner dark and well-thermoregulated cavity of the hive, preventing access to the less regulated parts of the hive (for example, next to the hive entrance or in the hive periphery). These nurse-age bees showed a similar phase to that of nurses in both trials (using all three phase indices; Supplementary Tables 10 and 11, Fig. 5c and Supplementary Fig. 5b). Their phase of activity differed from their sisters caged outside the hive in only one of the two trials (with colony 13–13). In the third trial with colony 13–10, the phase of activity for the caged nurse-age bees (‘Nurse-age, lab cage') was delayed relative to the LD cycle and the phase of the nurse age bees caged in the hive (‘Nurse-age, hive cage') appears between that of the nurses and the laboratory caged nurse-age bees. Nevertheless, as in the other trial (with colony 13–13; Fig. 5c), the phase of the nurse-age bees caged in the hive was not statistically different from that of free ranging nurses. These results show that entrainment by the colony environment can surpass photic entrainment of locomotor activity rhythms, even for young bees that are restricted to the inner parts of the hive and cannot directly sense ambient fluctuations in temperature or light.

## Discussion

By studying a massive data set obtained from experiments with >1,000 individuals, we demonstrate that social entrainment in honeybees is potent, can act without direct contact and is not mediated by gating photic input to the circadian clock. Entrainment by the social environment of the colony was robust and stable for several days even for 2-day-old bees, which are typically active around the clock with no overt circadian rhythms and show relatively weak entrainment by light. Remarkably, young bees that experienced conflicting photic and social environmental cycles showed a phase that was more similar to the social cycle, indicating that in honeybees social entrainment can override potent photic entrainment. Our findings are not limited to certain genotypes or laboratory lines, because we repeated each experiment two to four times, each with bees from a different source colony (which were genetically different). Our experimental protocol was specifically designed to clearly distinguish entrainment from masking, because we determined the circadian phase for bees transferred to individual cages in the constant laboratory environment and not while experiencing possible social and environmental cycles in the hive that could mask the influence of the internal clock. This study provides the strongest available evidence for the power of social entrainment and emphasizes the importance of studying circadian rhythms in a species-specific ecologically relevant context.

Nurse bees (that is, bees that typically care for the brood around the clock) in self-sustained, field-foraging colonies showed strong circadian rhythms in locomotor activity and a coherent phase when removed from constantly dark hives to individual cages in the laboratory (Fig. 1 and Supplementary Fig. 1). These experiments complement earlier studies showing strong synchronization for nurses that experienced LD cycles in observation hives^31^,^34^ and suggest that nurse bees in the hive are entrained by time-givers other than light. Quite surprisingly, similarly strong synchronization was also found for callow bees that were monitored after experiencing the colony environment for only the first 2 days of their life as adults (Fig. 2 and Supplementary Fig. 2). At this age, worker honeybees typically show low levels of activity and are mostly engaged in cleaning honeycomb cells^35^. Callow bees that were monitored individually at the same age, but experienced the hive environment for only 24 h, did not show similar strong phase coherence, suggesting that their clock is not yet fully developed, that more than 24 h are needed for effective entrainment by the colony environment or a combination of these two factors. Eban-Rothschild *et al*.^36^ have indeed shown that the colony environment is necessary for the development of circadian rhythms in callows, and that 24 h inside the colony are not sufficient for them to develop a rhythm. Additional studies in which bees at various ages are introduced to the hive for 24 or 48 h could clarify this ambiguity. The strong phase coherence was not compromised in 48-h-old bees that were caged in mesh enclosures in the centre of the brood area (Fig. 4 and Supplementary Fig. 4), indicating that entrainment in the hive does not require exposure to light at the hive entrance or to other microenvironmental cycles in peripheral parts of the hive that are less well regulated. Given that the hive cavity is constantly dark, and that the microenvironment in the brood area is tightly regulated^37^, we conclude that the synchronization of young nest bees in a colony is mediated by social factors.

Previous studies showed that honeybees that are confined in groups outside the colony can synchronize to a similar phase^38^,^39^. Are similar social interactions inside a hive sufficient to account for the strong entraining power of the colony? We addressed this question by comparing the synchronization of bees in the colony and in small groups in cages outside the hive. The laboratory caged bees showed weak phase coherence and their phase commonly differed from the ambient day–night cycle. Nevertheless, the phase coherence for the group-caged bees was typically stronger than in individually isolated bees from the same colony (Fig. 3 and Supplementary Fig. 3). These findings support and extend the earlier evidence for social entrainment in groups of honeybees. The phase coherence of bees in small groups was nonetheless weaker than for bees caged in similar groups inside the hive (Fig. 4a and Supplementary Fig. 4b). These results indicate that the colony environment is a stronger synchronizer than the sum of mutual interactions between 30 bees. Additional studies in which larger groups of bees are caged outside the hive are necessary, to fully understand the influence of worker number on social synchronization.

Light is considered the dominant time-giver responsible for the entrainment of circadian rhythms and the only one for which detailed characterization of the molecular and neuronal input pathways is available for both insects and vertebrates^40^,^41^,^42^. Honeybees, similar to other insects, are strongly entrained by light^43^,^44^,^45^. Indeed, photic entrainment was shown in this study for young bees that were caged in small groups (for example, see Fig. 5 and Supplementary Fig. 5). Individually isolated newly emerged bees can be also entrained by LD^43^,^46^, but in comparison with the strong and stable entrainment of young bees in the current study, the photic entrainment in the earlier studies appears weaker, because many newly emerged bees that experienced LD illumination regimes for 3 or 6 days in isolation lost circadian rhythms when relaeased to constant darkness (DD). Is it possible that social entrainment is stronger than photic entrainment? We explicitly addressed this question by subjecting nurse-age bees in freely foraging colonies to conflicting social and LD cycles. Young nest bees that experienced conflicing cycles of LD illumination and colony foraging activity cycles showed a phase that is more similar to foragers than to bees of a similar age that experienced the LD but not the social cycles (Fig. 5 and Supplementary Fig. 5). The entrainment by forager activity cycles rather than by the LD illumination regime was also seen for nurse-age bees that were caged in the inner parts of the hive. These findings rule out the possibility that the young nest bees are entrained by exposure to environmental time givers, for example, when flying out for ‘orientation flights'^47^, or when approaching the hive entrance. Thus, for the first time we show that social entrainment can override potent photic entrainment.

Our findings on the importance of social synchronization in honeybees set the stage for addressing a set of follow-up questions. For example, what are the social signals that mediate social synchronization? Our findings suggest that direct contact (for example, via contact pheromones or tactile communication) is not necessary for social entrainment in the hive. Temperature also seems unlikely, because the brood area is tightly thermoregulated; however, previous studies have shown that temperature cycles with an amplitude of 6–10 °C are needed for stably entraining circadian rhythms in honeybees^48^,^49^. Volatile pheromones, hive odours, vibrations or changes in the microenvironment (for example, CO_2_ concentration and humidity) seem to be more promising. Additional important lines for future inquiry include research on the sensory modalities perceiving the signals of social entrainment and the neuronal pathways transmitting the signal from sensory systems to the circadian clock system.

Another question raised by our findings is why do young nest bees that typically care for brood around the clock in a constant laboratory environment need to be entrained to the day–night cycles outside? Nest bees may need entrained circadian clocks to organize behaviours such as orientation flights^47^ and for timing interactions with bees performing outside tasks (for example, pollen and nectar flow into the hive during the day). A ticking circadian clock in around-the-clock active bees is perhaps also important for the coordination of internal metabolic processes (that is, intrinsic adaptive value^5^,^6^). The notion that circadian coordination of internal processes is important is supported by evidence that in diverse organisms disturbances to circadian functions compromise biochemical and metabolic processes, and are associated with a variety of diseases^50^,^51^. The hypothesis that some internal processes are circadianly regulated in around-the-clock active nest bees is also supported by evidence that many brain transcripts show circadian oscillations in nurse bees in the hive^52^. Social entrainment thus provides a powerful and efficient means to allow bees dwelling in the dark and constant environment of the nest to precisely adjust their behaviour and physiology to environmental cycles.

The potent social synchronization we show here for honeybees suggests that social signals may also be important time-givers for the clocks of other animals and should be taken into account in studies on animal ecology and chronobiology. Our field study suggesting the supremacy of social over photic entrainment for honeybees in a colony adds to recent studies with both mice and *Drosophila* showing the complexity of clock regulation in the real world. Under ecologically relevant conditions, photic entrainment may be less important than expected based on laboratory studies^53^,^54^,^55^. Taken together, these studies indicate that to understand how the circadian clock organizes behaviour and its functional significance, it is imperative to study animals in their species-specific natural context.

## Methods

### General design

Honeybee colonies were kept according to standard beekeeping techniques in the Bee Research Facility at the Edmond J. Safra campus of the Hebrew University of Jerusalem, Givat-Ram, Jerusalem, Israel. We identified foragers as bees returning to the hive with pollen loads on their hind legs. To obtain newly emerged bees, we removed honeycomb frames with sealed brood from field colonies, brushed off all adult bees and immediately placed the frame inside a lightproof box. We brought the box with the frame to the laboratory and placed it in an incubator (33±1 °C, 60% relative humidity (RH)). We collected adult bees emerging from the honeycomb within 30 min (Experiments 2–5) or 24 h (Experiments 1 and 6) of comb brushing. We then paint marked the callow bees on their thorax under dim red light and assigned the painted bees randomly to one of the experimental conditions as detailed for each experiment below. In Experiments 2–5 the bees that were introduced to field colonies were exposed to daylight twice (during the introduction and the collection from the hive); thus, we also exposed the bees from the other treatments to a similar daylight experience. The light exposure lasted less than a minute during the introduction to the field colony and 5–10 min during collection. The findings that the phase of bees isolated individually from these experiments was not synchronized to the subjective day (see Results) indicate that these two exposures to daylight, as well as the handling procedures, did not entrain the circadian clock of the bees. In Experiments 1 and 6, which were performed in indoor observation hives, the young bees were not exposed to light during the collection from the hive. Callow bees were randomly assigned to the different treatments. After being subjected to the different experimental conditions, the focal bees in all experiments were transferred to individual cages in a tightly regulated environmental chamber (constant dim red light, 29±1 °C, 60% RH) and their locomotor activity was monitored for 4–7 days (for example, see Fig. 1a). The locomotor activity data were analysed to determine various parameters related to circadian rhythms and social synchronization (see below).

### Observation hives

In Experiments 1 and 6, we established triple-cohort colonies with newly emerged bees (0–24 h of age), nurses, foragers (each cohort consisted of >1,500 bees) and their mother queen, all collected from the same source field colony (so all individuals were genetically related). To collect the cohort of foragers we blocked the entrance to the source field colony using a metal 8-hole per inch mesh and caught >1,500 foragers returning to the hive with pollen loads on their hind legs. We collected a similar number of nurse bees that were observed inserting their head into brood-containing cells. Newly emerged bees were collected as described above over a period of 2–4 successive days until reaching the desired number of >1,500 bees. Approximately 500 of the newly emerged bees were marked with a paint dot on their thorax under dim red light in all trials for Experiment 1 and trials 3 and 4 for Experiment 6, or under fluorescent light in trials 1 and 2 for Experiment 6. Each colony was housed in a two-frame observation hive (with transparent plexiglas walls, such that light could reach inside the hive) in an environmental chamber (29±1 °C; RH: 50±5%). The chamber was lit by constant dim red light (DD; using Edison Federal EFEE 1AE1 Deep Red LED; mean wavelength=660 nm, maximum and minimum wavelengths=670 and 650, respectively). The lower frame contained pollen and honey, and the upper frame had eggs and empty cells for the queen to lay. The observation hive was connected to the outside with a flexible plastic tube (length: 60 cm, inner diameter=28 mm). The plastic tube was wrapped with opaque aluminum foil and was curved in an S-shape, preventing the exposure of the inner parts of the colony to outside light. The hive entrance was controlled by a plastic sliding gate.

### Analyses of circadian rhythms for individual bees

Locomotor activity for individual bees was monitored with the ClockLab data acquisition system^31^,^56^. We placed each bee individually into a monitoring cage made from a modified Petri dish (diameter=90 mm), provisioned with *ad libitum* sugar syrup (50% w/w) and without pollen. We placed the monitoring cages with the focal bees into an environmental chamber (29±1 °C; RH=45±5%), in constant dim red light (Edison Federal EFEF 1AE1 Far (Cherry) Red LED; mean wavelength=740 nm, maximum and minimum wavelengths were 750 and 730, respectively). Data were collected continuously at a frequency of 1 Hz with four charge-coupled device cameras (Panasonic WV-BP334) and an image acquisition board (IMAQ 1,409, National Instruments, USA). For the analyses of circadian rhythms, we used the ClockLab circadian analyses software (Actimetrics, USA). The time at onset and offset of activity was defined as the beginning and end, respectively, of the daily bout of elevated locomotion. The precise timing of the onset or offset were defined as at least three consecutive 10-min bins each with activity reaching at least 10% of the maximum activity per bin during this day. In addition, there needed to be a period of at least 5 h of reduced activity between the offset and the following onset. We fitted linear regression lines passing through both the onset and offset of activity during the first 4 days in the laboratory (Fig. 1b). We used the extrapolations of these regression lines on the day of transfer to the laboratory for estimating the timing of onset and offset of activity (which served as indices for phase). The median time of activity was calculated as the midpoint between the times of onset and offset of activity. We used only bees for which we could unambiguously determine the onset/offset of the daily activity bout and omitted those that did not show significant (*P*<0.01) rhythmicity in a *χ*^2^-periodogram analysis^57^. Actograms were analysed blindly in regards to treatment. We used the Oriana software package (KCS, USA) for circular statistical analyses. We used the Rayleigh test and mean length of vector as an index for phase coherence, and the Watson–Williams F-test for comparing the phase of two or more groups (with Bonferroni *post-hoc* tests). For each bee we also determined the ‘*α*', which is the time between the onset and offset of activity.

### Phase synchronization for nurses and foragers

In Experiment 1 we compared forager bees that experienced 12 h light/12 h dark (LD) cycles and nurses that experienced a constantly dark and thermoregulated hive. We established triple-cohort colonies in observation hives as described above and kept them under constant dim red light until day 8. The hive entrance was opened at 0800, h and closed at 2000, h. Thus, the foragers experienced light during the day when flying outside and darkness during the night, while nurses spent most, if not all, of their time in the inner dark cavity of the hive. On day 8, we observed the hive entrance and paint-marked a few hundred foragers. On the same day, we collected the marked nurses (now 7–8 days of age) in DD and the marked foragers in LD, transferred them to individual cages and monitored their locomotor activity under constant laboratory conditions. We repeated the experiment two times, each with bees from a different source colony (H7 and S8).

### Phase synchronization for callow bees in a field colony

In the second experiment (Experiment 2) we tested whether nest bees younger than the typical age of nurses can be also synchronized by the colony environment. We assigned newly emerged bees to one of the following treatments: (i) 48 h in a field colony (‘Colony 48 h'); (ii) 24 h in a field colony (‘Colony 24 h'); (iii) 48 h isolated in a Petri dish provisioned with sugar syrup (50% w/v) and pollen, and placed in the laboratory (31±1 °C; RH=55±5%, ‘Lab individually 48 h'). As a positive control we collected foragers, which typically exhibit strong circadian rhythms^30^,^58^. After 24 or 48 h under the different experimental conditions, we collected the young bees and the foragers from the hive and transferred them to the locomotor activity monitoring cages in the lab. We transferred the individually isolated bees to new monitoring cages. We repeated the experiment three times, each with bees from a different source colony (H11, S73 and H14).

### Synchronization of bees caged in groups outside the hive

In Experiment 3, we assigned newly emerged bees to one of the following treatments: (i) 48 h in a field colony (‘Colony 48 h'), (ii) 48 h in a wooden cage (11 × 10 × 4.5 cm) with 30 same-age sister bees in the laboratory and provisioned with *ad libitum* pollen and sugar syrup (‘Lab group 48 h') and (iii) 48 h individually isolated in cage in the laboratory (‘Lab individually 48 h', similar to group (iii) in Experiment 2. We repeated the experiment three times, each with a different source colony (H6, H12 and H11).

### The importance of contact with other bees in the hive

In Experiment 4, we assigned newly emerged worker bees to one of the following treatments: (i) 48 h in a field colony (‘Colony 48 h'), (ii) 48 h in a SM enclosure with 30 same-age sister bees inside the same field colony (‘Colony SM'), (iii) 48 h in a DM enclosure containing 30 same-age sister bees inside the colony (‘Colony DM') and (iv) 48 h in a wooden cage with 30 same-age sister bees in the laboratory (‘Lab group 48 h'). The enclosures (11 × 10.5 × 2 cm) were made of an 8-hole per inch mesh and were embedded on both sides of an empty comb. For the DM treatment, the enclosure was fenced by a larger 8-hole per inch mesh cage (14 × 13.5 × 3 cm) such that the gap between the two meshes was 1.5 cm. Both the SM and DM enclosures prevented the caged bees from interacting with the brood but allowed exposure to the microenvironment (for example, temperature, humidity and CO_2_ levels), as well as to the hive odours. Only the DM caging prevented direct contact with bees outside the enclosure. We placed the frame with the focal bees in the centre of the hive, such that the caged bees were flanked by brood-containing honeycombs. Group size and density was similar for the Lab group 48 h, Colony SM and Colony DM treatments. We provisioned all cages (inside and outside the colony) with sugar syrup and pollen. We repeated this experiment three times, each with bees from a different source colony (H2, HS76 and S85).

Given that bees inside the mesh enclosure can entrian each other, we performed an additional experiment (Experiment 5) with individully caged bees. We assigned newly emerged bees to one of the following treatments: (i) 48 h in a field colony (‘Colony 48 h'), (ii) caged individually in a SM (8-hole per inch, same as above) enclosure in the same colony for 48 h (‘Isolated SM'), (iii) caged individually in a DM enclosure in the same colony for 48 h (‘Isolated DM') and (iv) 48 h in an individual cage (7.5 × 2.5 × 2.5 cm) in the laboratory (‘Lab individually 48 h'). The side walls of the individual cages of this treatment were made of transparent glass. The lid (2.5 × 2.5) was made of 8-holes per inch mesh, allowing ventilation. As in this experiment we placed the individual glass cages one next to the other we cannot exclude the possibility that the bees sensed olfactory or vibratory cues emitted from neighbouring cages.

For caging bees in individual enclosures inside the hive, we constructed a horizontal wooden separation in the centre of an empty honeycomb frame (without a comb) that divided the frame into two similar compartments (10 × 3 × 43.4 cm). In each compartment we placed a row of 11 mesh cages (7.5 × 2.5 × 2.5 cm), with a gap of 1.5 cm between each pair of adjacent cages. For the DM treatment, we covered the lower part of the frame with an additional mesh placed at least 1.5 cm apart from the SM enclosures. In each trial we introduced 11 bees for each of the treatments (33 bees in total). All the focal bees were sisters obtained from the same source colony. We placed the frames with the cages in the centre of the hive, such that they were flanked by brood-containing honeycomb frames. We provisioned all cages (inside and outside the hive) with *ad libitum* sugar syrup and pollen. We repeated this experiment two times, each with bees from a different source colony (H11 and H2).

### Entrainment with conflicting photic and social time-givers

The general outline of this experiment (Experiment 6) is described in Fig. 5a. We performed four trials, each with a different triple cohort colony (see above). The first two trials included three treatments: (i) ‘Nurse': paint marked newly emerged bees that were introduced into the hive and later collected after being observed nursing brood; (ii) ‘Nurse-age, lab cage': newly emerged bees that were introduced into the hive for 2 days and then, on day 3, transferred to a wooden cage (4 × 10 × 4.5 cm) that was placed next to the observation hive in the same environmental chamber. The cage was placed on a styrofoam base to prevent transfer of vibrations from the observation hive to the cage. (iii) ‘Forager': bees of unknown age that returned to the hive with conspicuous pollen loads in their corbiculae. Starting on day 1, we opened the hive entrance every day for 8 h, such that foragers were able to collect food and water and to be exposed to sunlight on foraging trips during the 8 h of hive opening (opened: 1200 or 1000, h, closed: 2000 or 1800, h in Trial 1 (colony J12) and Trial 2 (colony O12), respectively). We continued with this hive opening/closing schedule for the next 7 days until day 8. Starting on day 3, we also exposed the hive to a LD illumination regime that was in an advanced phase compared with the opening and closing schedule (lights-on (200 lux): 0600 or 0400, h; lights-off: 1400 or 1200, h, in Trials 1 and 2, respectively). Thus, during days 3–8, the lights were turned on and off 6 h before the hive opening and closing. On day 8, when the nurses and nurse-age bees were 7–8 days of age, we transferred focal bees from all three treatment groups to individual monitoring cages in constant laboratory conditions and monitored their locomotor activity for 5 days. The bees from the first two groups were collected in the environmental chamber under dim red light.

In the last two trials (3 and 4) of this experiment we added an additional fourth treatment consisting of newly emerged bees that were caged in a SM enclosure (with dimensions similar to the ‘lab cage') inside the hive (‘Nurse-age, hive cage'; Supplementary Fig. 6). The hive entrance was opened at 1100 or 1030, h and closed at 1900 or 1830, h, in Trial 3 (colony 13–10) and Trial 4 (colony 13–13), respectively. The illumination regime was lights-on: 0500 or 0430, h, lights-off: 1300 or 1230, h, in Trial 3 (colony 13–10) and Trial 4 (colony 13–13), respectively. Each trial was performed with bees from a differenct source colony.

### Data availability

All data supporting the findings of this study are available within the article, its Supplementary Information files, or from the corresponding authors on request.

## Additional information

**How to cite this article:** Fuchikawa, T. *et al*. Potent social synchronization can override photic entrainment of circadian rhythms. *Nat. Commun.* 7:11662 doi: 10.1038/ncomms11662 (2016).

## Supplementary Material

Supplementary Information Supplementary Figures 1-6 and Supplementary Tables 1-11
