# Supplementary material for: Potent social synchronization can override photic entrainment of circadian rhythms
Source: Nat Commun. 2016 May 23;7:11662. doi: 10.1038/ncomms11662 (PMC4879263; doi:10.1038/ncomms11662)
Supplement: Supplementary Information — Supplementary Figures 1-6 and Supplementary Tables 1-11 [file ncomms11662-s1.pdf]

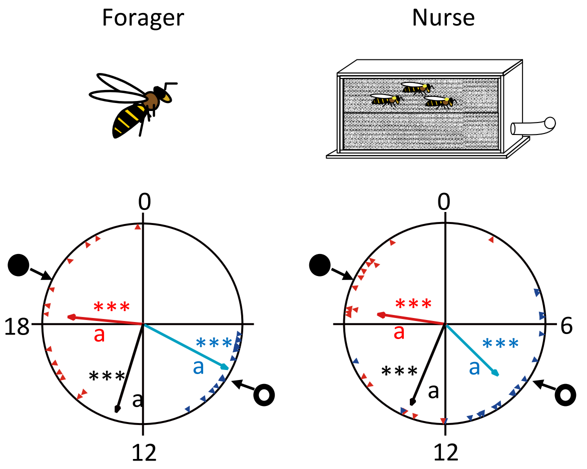

Supplementary Figure 1. Nurses removed from colonies foraging–in the field are synchronized to the day-night cycle outside. A repetition with bees from colony S8. The time of day is depicted on the circular plot perimeter. The open and filled circles on the perimeter delineate the time of sunrise and sunset, respectively. The blue and red triangles depict the time of onset and offset of activity for individual bees, respectively. The blue, black and red vectors (arrow) pointing from the center toward the perimeter show the average time for the onset, median, and offset, respectively. Vector length corresponds to the extent of phase coherence. Asterisks in matching colors correspond to the p-value obtained from a Rayleigh test for phase coherence (*: 0.01<p<0.05, **: 0.001<p<0.01, ***: p<0.001). The median for each bee (points for individual bees are not shown) was calculated as the midpoint between the onset and offset. Vectors sharing the same color across different plots, and which are marked with different small letters, are significantly different in a Watson-Williams F- test across experimental groups. Sample sizes are 13 and 18, for forager and nurse, respectively.

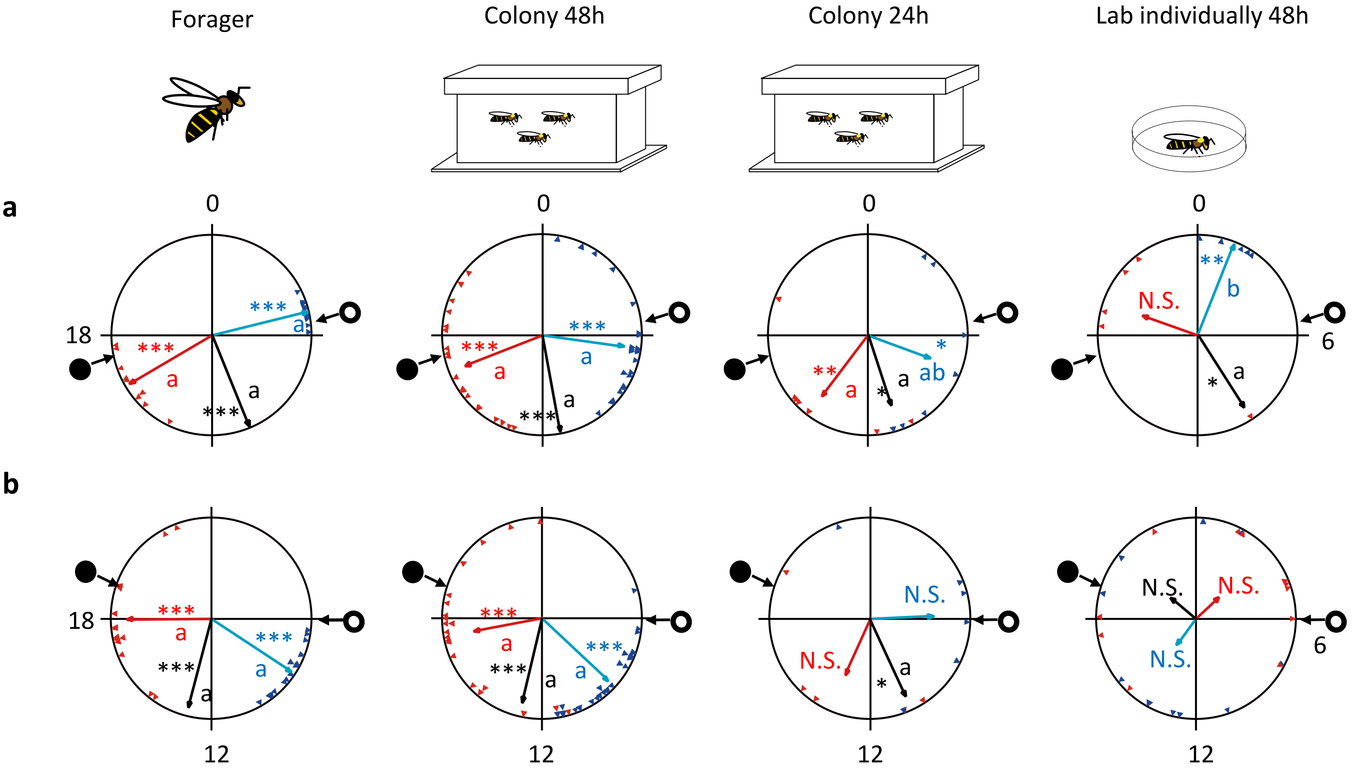

Supplementary Figure 2. Forty-eight hours in the hive are sufficient for strong synchronization of newly emerged bees to the colony phase. (a) A repetition with bees from colony S73. (b) A repetition with bees from colony H14. The open and filled circles on the perimeter delineate the time of sunrise and sunset, respectively. The blue and red triangles depict the time of onset and offset of activity for individual bees, respectively. The blue, black and red vectors (arrow) pointing from the center toward the perimeter show the average time for the onset, median, and offset, respectively. Vector length corresponds to the extent of phase coherence. Asterisks in matching colors correspond to the p-value obtained from a Rayleigh test for phase coherence (*: $0.01<p<0.05$, **: $0.001<p<0.01$, ***: $p<0.001$). The median for each bee (points for individual bees are not shown) was calculated as the midpoint between the onset and offset. Vectors sharing the same color across different plots, and which are marked with different small letters, are significantly different in a Watson-Williams F- test with Bonferroni post hoc tests across experimental groups. 'Colony 48h' – young bees that experienced their first 48 h in a field colony and then monitored individually in constant laboratory conditions. 'Colony 24h'- same as above, but bees experienced the colony environment for only 24 hrs. 'Lab individually 48h' - young bees that experienced their first 48 h isolated individually and then monitored individually in constant laboratory conditions. Sample sizes are 11, 23, 7, 5 for ‘Forager’, ‘Colony 48h’, ‘Colony 24h’, ‘Lab individually 48h’, respectively for colony S73, and15, 24, 6, 11, for colony H14.

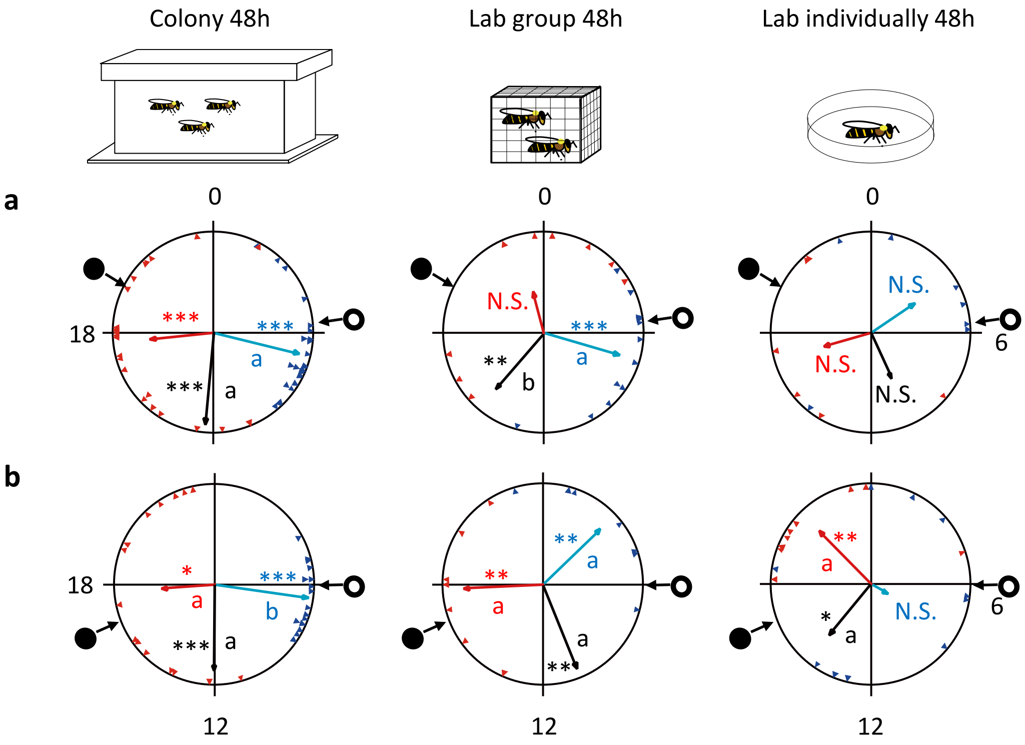

Supplementary Figure 3. Social interactions with 30 bees produce weaker synchronization than the hive environment. (a) A repetition with bees from colony H12. (b) A repetition with bees from colony H11. The open and filled circles on the perimeter delineate the time of sunrise and sunset, respectively. The blue and red triangles depict the time of onset and offset of activity for individual bees, respectively. The blue, black and red vectors (arrow) pointing from the center toward the perimeter show the average time for the onset, median, and offset, respectively. Vector length corresponds to the extent of phase coherence. Asterisks in matching colors correspond to the p-value obtained from a Rayleigh test for phase coherence (*: 0.01<p<0.05, **: 0.001<p<0.01, ***: p<0.001). The median for each bee (points for individual bees are not shown) was calculated as the midpoint between the onset and offset. Vectors sharing the same color across different plots, and which are marked with different small letters, are significantly different in a Watson-Williams F- test with Bonferroni post hoc tests across experimental groups. 'Lab group 48h' - young bees that experienced their first 48 h in a cage with 30 other same-age bees before being monitored individually in constant laboratory conditions. Sample sizes are 24, 12, 7 for ‘Colony 48h’, ‘Lab group 48h’, ‘Lab individually 48h’, respectively for colony H12, 16, 7, 10, for colony H11.

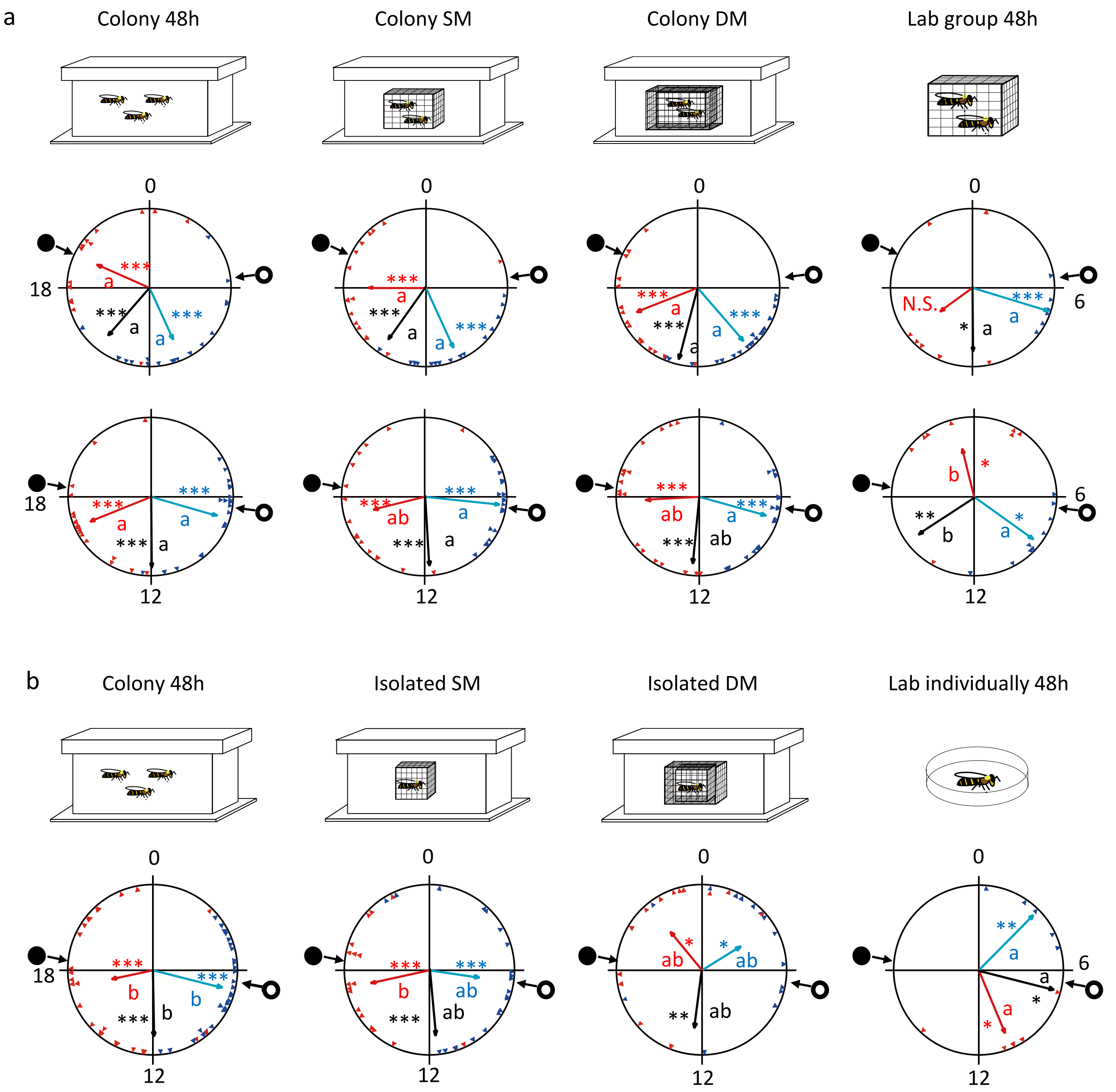

Supplementary Figure 4. Direct contact with other bees is not needed for entrainment by the colony environment. (a) Circular statistics for bees from Experiment 4 in which bees were caged in groups. Upper row, a repetition with bees from colony HS76; lower row, a repetition with bees from colony S85. Asterisks in matching colors correspond to the p-value obtained from a Rayleigh test for phase coherence (*: $0.01<p<0.05$, **: $0.001<p<0.01$, ***: $p<0.001$). The median for each bee (points for individual bees are not shown) was calculated as the midpoint between the onset and offset. Vectors sharing the same color across different plots, and which are marked with different small letters, are significantly different in a Watson-Williams F- test with Bonferroni post hoc tests across experimental groups. Sample sizes are 19, 24, 21, 8 for ‘Colony 48h’, ‘Colony SM’, ‘Colony DM’, ‘Lab individually 48h’, respectively for colony HS76. 26, 23, 24, 14, for colony S85 (b) Circular statistics for bees from Experiment 5 in which bees from colony H2 were caged individually. ‘Isolated–SM' and Isolated DM‘, same as in ‘Colony SM’, and ‘Colony DM’ above (respectively), but bee was caged individually. Other details as in (a). Sample sizes are 29, 20, 14, 5, for ‘Colony 48h’, ‘Isolated SM’, ‘Isolated DM’, ‘Lab individually 48h’, respectively.

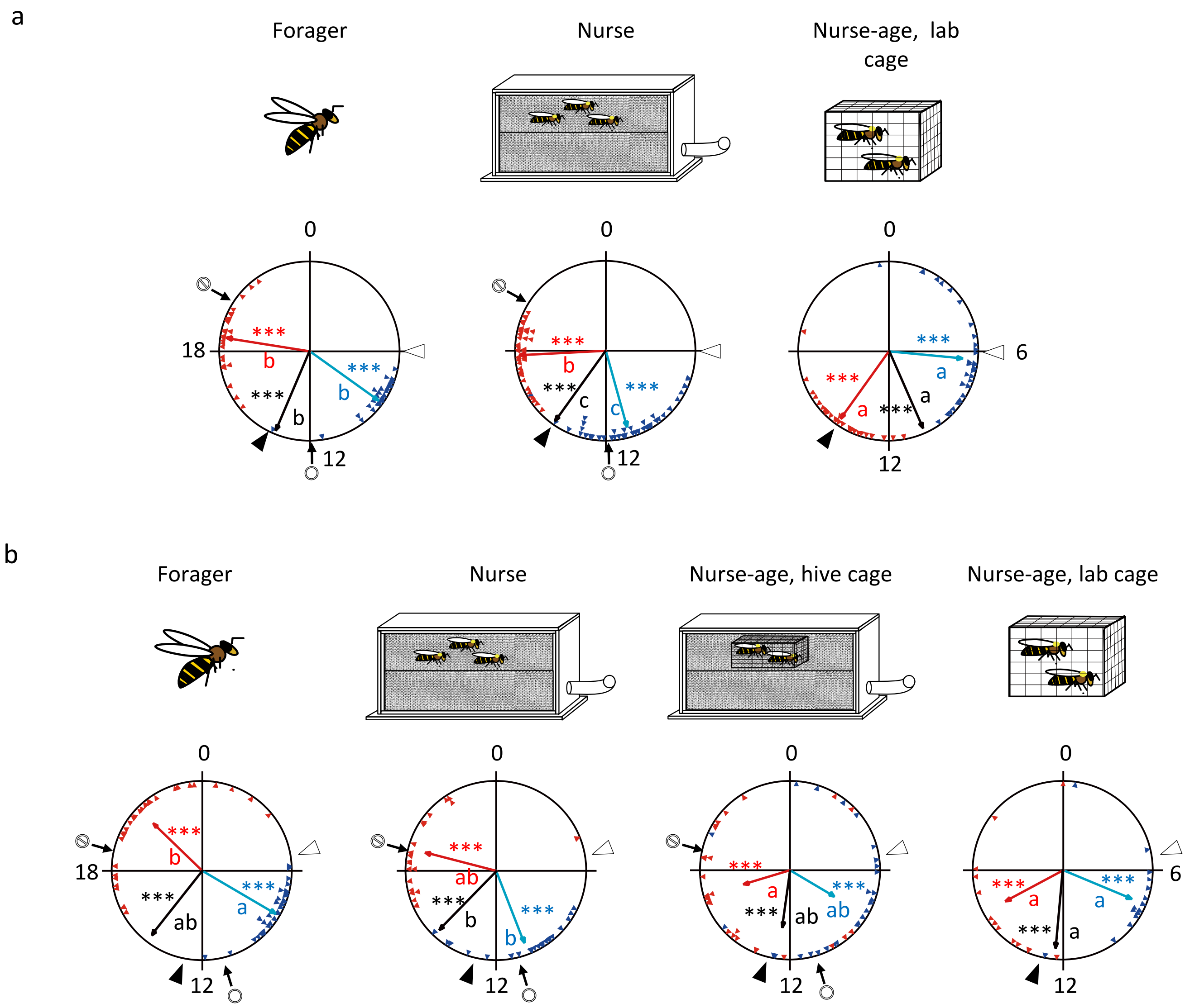

Supplementary Figure 5. Entrainment in bees experiencing conflicting photic and social time givers. (a) Circular statistics for Trial 1 with bees from colony J12. The circle and the circle-backslash symbols point to the times of opening and closing the hive entrance, respectively. Open and filled arrowheads point to the times of light-on and -off in the chamber in which the observation hive and cage were housed. The blue and red triangles depict the time of onset and offset of activity for individual bees, respectively. The blue, black and red vectors (arrow) pointing from the center toward the perimeter show the average time for the onset, median, and offset, respectively. Vector length corresponds to the extent of phase coherence. Asterisks in matching colors correspond to the p-value obtained from a Rayleigh test for phase coherence (*: 0.01<p<0.05, **: 0.001<p<0.01, ***: p<0.001). The median for each bee (points for individual bees are not shown) was calculated as the midpoint between the onset and offset. Vectors sharing the same color across different plots, and which are marked with different small letters, are significantly different in a Watson-Williams F- test with Bonferroni post hoc tests across experimental groups. 'Nurse-age, lab cage' – a group of 30 newly emerged bees were housed in a cage that was placed next to the observation hive, and experienced only the light-dark cycles. The bees from all groups were transferred to locomotor activity monitoring cages at the age of 8 days. Sample sizes are 26, 39, 30 for ‘Forager’, ‘Nurse’, ‘Nurse-age, lab cage’, respectively. (b) Circular statistics for Trial 3 with bees from colony 13-10. This trial had an additional treatment that consisted of newly emerged bees that were caged in a single mesh enclosure inside the hive ('Nurse-age, hive cage'). Sample sizes are 30, 21, 26, 21, for ‘Forager’, ‘Nurse’, ‘Nurse-age, hive cage’, ‘Nurse-age, lab cage’, respectively.

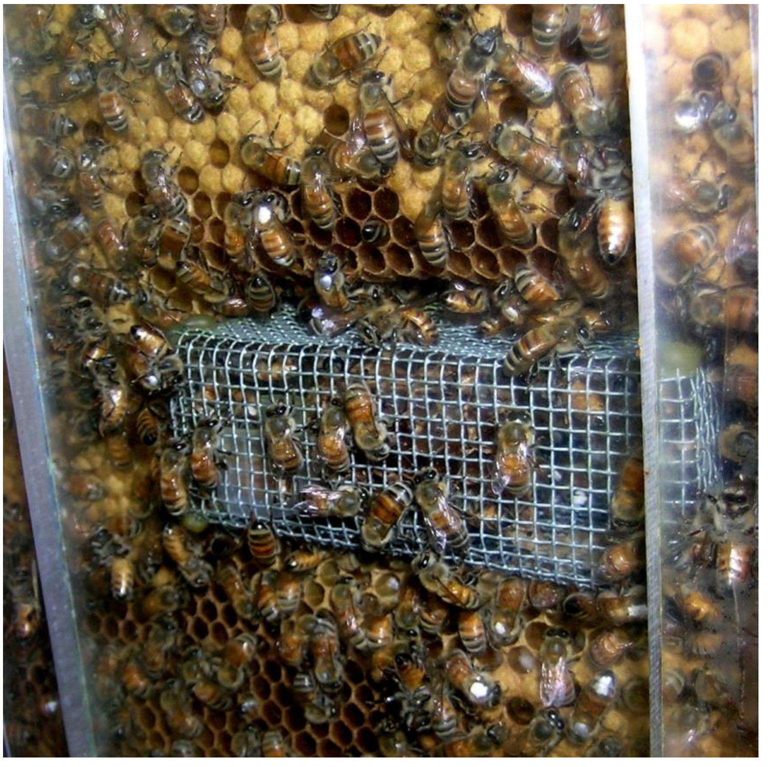

Supplementary Figure 6. A single-mesh enclosure that was used to restrict bees to the inner part of the hive in Experiment 6. The enclosure was made from a folded 8-hole per inch mesh. Bees inside the enclosure were provisioned with 50 % (w/v) sugar water and a pea-sized ball of pollen.

Supplementary Table 1. A summary of circular statistics for Experiment 1

		Colony H7		Colony S8	
		**Forager**	**Nurse**	**Forager**	**Nurse**
**Onset**	Sample size (n)	11	17	13	18
	Mean vector (h:min)	7:55a	10:40b	7:50a	8:56a
	Length of mean vector (r)	0.990	0.847	0.955	0.732
	Rayleigh test (p)	<0.001	<0.001	<0.001	<0.001
**Median**	Sample size (n)	11	15	13	17
	Mean vector (h:min)	13:42a	13:36a	13:08a	13:29a
	Length of mean vector (r)	0.965	0.939	0.911	0.870
	Rayleigh test (p)	<0.001	<0.001	<0.001	<0.001
**Offset**	Sample size (n)	11	15	13	17
	Mean vector (h:min)	19:28b	16:50a	18:20a	18:32a
	Length of mean vector (r)	0.874	0.816	0.744	0.673
	Rayleigh test (p)	<0.001	<0.001	<0.001	<0.001
**Alpha**	(h:min)	11:36	6:33	10:36	9:46

Supplementary Table2. A summary of circular statistics for Experiment 2

		**Colony S73**				**Colony H14**				**Colony H11**			
		**Forager**	**Colony 48h**	**Colony 24h**	**Lab individually 48h**	**Forager**	**Colony 48h**	**Colony 24h**	**Lab individually 48h**	**Forager**	**Colony 48h**	**Colony 24h**	**Lab individually 48h**
**Onset**	Sample size (n)	11	23	7	5	14	24	6	11	15	17	13	11
	Mean vector (h:min)	5:05a	6:31a	7:21ab	1:27b	8:14a	8:53a	(5:51)	(14:26)	6:11a	6:42a	(4:58)	(8:43)
	Length of mean vector (r)	0.994	0.830	0.666	0.977	0.960	0.905	0.636	0.334	0.977	0.942	0.468	0.496
	Rayleigh test (p)	<0.001	<0.001	0.039	0.002	<0.001	<0.001	0.084^{NS}	0.3^{NS}	<0.001	<0.001	0.055^{NS}	0.064^{NS}
**Median**	Sample size (n)	9	19	6	5	14	21	5	11	14	16	13	11
	Mean vector (h:min)	10:30a	11:40a	10:48a	9:50a	12:57a	12:54a	10:21a	(20:35)	12:00ab	10:46a	9:28a	14:48b
	Length of mean vector (r)	0.987	0.892	0.734	0.850	0.939	0.853	0.879	0.346	0.929	0.886	0.643	0.639
	Rayleigh test (p)	<0.001	<0.001	0.032	0.018	<0.001	<0.001	0.012	0.274^{NS}	<0.001	<0.001	0.003	0.008
**Offset**	Sample size (n)	9	19	7	5	15	21	5	11	15	16	13	11
	Mean vector (h:min)	15:57a	16:32a	14:29a	(19:16)	17:58a	17:18a	(13:34)	(3:05)	17:38ab	14:51a	14:49a	20:58b
	Length of mean vector (r)	0.954	0.833	0.767	0.589	0.848	0.694	0.615	0.314	0.757	0.673	0.618	0.711
	Rayleigh test (p)	<0.001	<0.001	0.01	0.182^{NS}	<0.001	<0.001	0.153^{NS}	0.347^{NS}	<0.001	<0.001	0.008	0.002
**Alpha**	(h:min)	10:53	10:30	7:08	(16:25)	9:27	8:22	(7:43)	(15:09)	11:51	8:34	(9:51)	(12:24)
	% of rhythmic bees (number of tested bees)	100 (12)	86 (29)	37 (19)	28 (18)	100 (17)	90 (29)	50 (24)	75 (24)	100 (20)	95 (22)	79 (24)	67 (24)

Supplementary Table 3. A summary of vector length for Experiment 2

Length of mean vector (mean±SE, n=3)	**Forager**	**Colony 48h**	**Colony 24h**	**Lab individually 48h**
Onset	0.977±0.012	0.892±0.04	0.59±0.075	0.602±0.236
Median	0.952±0.022	0.877±0.015	0.752±0.084	0.612±0.179
Offset	0.853±0.07	0.733±0.061	0.667±0.061	0.538±0.144

Supplementary Table 4. A summary of circular statistics for Experiment 3

		Colony H12			Colony H6			Colony H11		
		**Colony 48h**	**Lab group 48h**	**Lab individually 48h**	**Colony 48h**	**Lab group 48h**	**Lab individually 48h**	**Colony 48h**	**Lab group 48h**	**Lab individually 48h**
**Onset**	Sample size (n)	24	12	7	21	11	7	16	7	10
	Mean vector (h:min)	6:54a	7:05a	(3:47)	7:52	(2:29)	(1:36)	6:33b	3:03a	(7:52)
	Length of mean vector (r)	0.881	0.792	0.527	0.900	0.488	0.256	0.948	0.788	0.199
	Rayleigh test (p)	<0.001	<0.001	0.144^{NS}	<0.001	0.07^{NS}	0.648^{NS}	<0.001	0.008	0.684^{NS}
**Median**	Sample size (n)	22	9	6	20	11	7	15	7	9
	Mean vector (h:min)	12:20a	14:42b	(10:21)	12:22a	11:30a	(9:26)	12:01a	10:30a	14:37a
	Length of mean vector (r)	0.913	0.707	0.501	0.910	0.666	0.496	0.844	0.878	0.641
	Rayleigh test (p)	<0.001	0.007	0.23^{NS}	<0.001	0.005	0.183^{NS}	<0.001	0.001	0.02
**Offset**	Sample size (n)	23	9	6	20	11	7	15	7	9
	Mean vector (h:min)	17:35	23:02	16:55	16:47a	19:51a	17:19a	17:41a	17:47a	21:03a
	Length of mean vector (r)	0.641	0.419	0.490	0.729	0.644	0.700	0.533	0.798	0.741
	Rayleigh test (p)	<0.001	0.209^{NS}	0.245^{NS}	<0.001	0.007	0.026	0.012	0.007	0.004
**Alpha**	(h:min)	11:03	(15:12)	(11:31)	8:52	16:27	(16:35)	11:07	14:45	(14:01)

Supplementary Table 5. A summary of vector length for Experiment 3

Length of vector (mean±SE, n=3)	**Colony 48h**	**Lab group 48h**	**Lab individually 48h**
**Onset**	0.910±0.024	0.689±0.123	0.327±0.124
**Median**	0.889±0.028	0.750±0.080	0.546±0.058
**Offset**	0.634±0.069	0.620±0.135	0.644±0.095

Supplementary Table 6. A summary of circular statistics for Experiment 4

		Colony HS76				Colony S85				Colony H2			
		**Colony 48h**	**Colony SM**	**Colony DM**	**Lab group 48h**	**Colony 48h**	**Colony SM**	**Colony DM**	**Lab group 48h**	**Colony 48h**	**Colony SM**	**Colony DM**	**Lab group 48h**
**Onset**	Sample size (n)	19	23	21	7	26	23	24	14	28	24	18	14
	Mean vector (h:min)	10:32a	9:45a	9:34a	7:39a	7:25a	6:42a	7:32a	7:18a	9:46a	10:52a	9:15a	9:48a
	Length of mean vector (r)	0.739	0.808	0.856	0.922	0.799	0.897	0.832	0.494	0.882	0.909	0.880	0.594
	Rayleigh test (p)	<0.001	<0.001	<0.001	<0.001	<0.001	<0.001	<0.001	0.03	<0.001	<0.001	<0.001	0.005
**Median**	Sample size (n)	15	15	15	6	18	16	19	8	20	17	7	10
	Mean vector (h:min)	14:39a	14:19a	12:58a	11:55a	11:59a	11:45a	12:21ab	15:41b	14:29a	14:45a	13:31a	(15:46)
	Length of mean vector (r)	0.792	0.802	0.925	0.812	0.901	0.879	0.843	0.820	0.875	0.909	0.916	0.502
	Rayleigh test (p)	<0.001	<0.001	<0.001	0.012	<0.001	<0.001	<0.001	0.002	<0.001	<0.001	<0.001	0.078^{NS}
**Offset**	Sample size (n)	17	24	17	8	24	18	21	12	27	21	13	11
	Mean vector (h:min)	19:37a	18:53a	16:58a	(16:24)	17:09a	17:11ab	17:56ab	21:51b	19:09a	18:30a	17:43a	(21:15)
	Length of mean vector (r)	0.608	0.628	0.787	0.490	0.791	0.650	0.652	0.534	0.713	0.846	0.783	0.439
	Rayleigh test (p)	0.001	<0.001	<0.001	0.148^{NS}	<0.001	<0.001	<0.001	0.029	<0.001	<0.001	<0.001	0.119^{NS}
**Alpha**	(h:min)	10:00	8:04	7:03	(9:46)	9:15	11:01	10:28	13:48	9:14	7:34	7:00	(11:51)

Supplementary Table 7. A summary of vector length for Experiment 4

Length of vector (mean±SE, n=3)	**Colony 48h**	**Colony SM**	**Colony DM**	**Lab group 48h**
**Onset**	0.807±0.051	0.871±0.039	0.856±0.017	0.670±0.158
**Median**	0.856±0.040	0.863±0.039	0.895±0.032	0.711±0.128
**Offset**	0.704±0.065	0.708±0.085	0.741±0.054	0.488±0.034

Supplementary Table 8. A summary of circular statistics for Experiment 5

		Colony H11				Colony H2			
		Colony 48h	Isolated SM	Isolated DM	Lab individually 48h	Colony 48h	Isolated SM	Isolated DM	Lab individually 48h
**Onset**	Sample size (n)	24	21	24	16	29	20	14	5
	Mean vector (h:min)	8:22a	8:14a	6:46a	11:03a	6:56b	6:32ab	3:54ab	2:59a
	Length of mean vector (r)	0.901	0.624	0.623	0.532	0.842	0.595	0.534	0.914
	Rayleigh test (p)	<0.001	<0.001	<0.001	0.009	<0.001	<0.001	0.016	0.007
**Median**	Sample size (n)	24	21	23	14	27	18	13	4
	Mean vector (h:min)	13:08a	13:02a	12:29a	14:20a	11:57b	11:39ab	12:28ab	6:56a
	Length of mean vector (r)	0.921	0.697	0.633	0.573	0.777	0.775	0.671	0.911
	Rayleigh test (p)	<0.001	<0.001	<0.001	0.008	<0.001	<0.001	0.002	0.024
**Offset**	Sample size (n)	25	22	24	15	27	18	13	5
	Mean vector (h:min)	17:37a	17:15a	18:10a	18:07a	17:11b	17:10b	21:21ab	10:30a
	Length of mean vector (r)	0.815	0.528	0.492	0.461	0.499	0.711	0.581	0.803
	Rayleigh test (p)	<0.001	0.002	0.002	0.039	<0.001	<0.001	0.01	0.03
**Alpha**	(h:min)	9:30	10:03	10:42	8:54	10:29	11:15	15:42	9:00

Supplementary Table 9. A summary of vector length for Experiment 5

Length of vector (mean±SE, n=2)	Colony 48h	Isolated SM	Isolated DM	Lab individually 48h
**Onset**	0.872±0.042	0.610±0.021	0.579±0.063	0.723±0.270
**Median**	0.849±0.102	0.736±0.055	0.652±0.027	0.742±0.239
**Offset**	0.657±0.223	0.620±0.129	0.537±0.063	0.632±0.242

Supplementary Table 10. A summary of circular statistics for Experiment 6

		Trial 1: J12			Trial 2: O12			Trial 3: Colony 13-10				Trial 4: Colony 13-13			
		**Forager**	**Nurse**	**Nurse-age, lab cage**	**Forager**	**Nurse**	**Nurse-age, lab cage**	**Forager**	**Nurse**	**Nurse-age, hive cage**	**Nurse-age, lab cage**	**Forager**	**Nurse**	**Nurse-age, hive cage**	**Nurse-age, lab cage**
**Onset**	Sample size (n)	26	39	30	34	35	24	30	21	26	21	15	28	22	21
	Mean vector (h:min)	8:23b	10:57c	6:22a	5:35b	7:29c	4:12a	8:00a	10:34b	8:00ab	5:04a	6:09ab	7:00bc	7:37c	5:04a
	Length of mean vector (r)	0.940	0.876	0.826	0.959	0.861	0.857	0.942	0.868	0.573	0.892	0.966	0.870	0.938	0.892
	Rayleigh test (p)	<0.001	<0.001	<0.001	<0.001	<0.001	<0.001	<0.001	<0.001	<0.001	<0.001	<0.001	<0.001	<0.001	<0.001
**Median**	Sample size (n)	26	39	24	34	34	22	30	20	26	21	13	28	22	21
	Mean vector (h:min)	13:31b	14:23c	10:24a	11:42b	11:47b	8:51a	14:30ab	14:55b	12:30ab	10:48a	14:11b	12:26ab	13:42b	10:48a
	Length of mean vector (r)	0.957	0.955	0.934	0.915	0.861	0.897	0.912	0.914	0.637	0.883	0.932	0.851	0.912	0.883
	Rayleigh test (p)	<0.001	<0.001	<0.001	<0.001	<0.001	<0.001	<0.001	<0.001	<0.001	<0.001	<0.001	<0.001	<0.001	<0.001
**Offset**	Sample size (n)	26	39	26	34	34	23	30	20	26	21	13	28	22	21
	Mean vector (h:min)	18:37b	17:48b	14:20a	17:42b	15:53b	12:59a	20:56b	18:56ab	16:53a	14:32a	20:06c	15:40ab	17:54bc	14:32a
	Length of mean vector (r)	0.928	0.951	0.919	0.724	0.757	0.782	0.760	0.814	0.543	0.689	0.847	0.695	0.672	0.689
	Rayleigh test (p)	<0.001	<0.001	<0.001	<0.001	<0.001	<0.001	<0.001	<0.001	<0.001	<0.001	<0.001	<0.001	<0.001	<0.001
**Alpha**	(h:min)	10:09	6:50	7:56	12:14	8:54	9:04	12:58	8:42	8:46	9:33	14:00	8:55	10:17	9:33
**Phase**, Median (normalized to Forager)		0:00	0:52	−3:07	0:00	0:05	−2:51	0:00	0:25	−2:00	−2:09	0:00	−1:45	−0:29	−3:23

Supplementary Table11. A summary of vector length for Experiment 6

Length of vector (mean±SE (n))	**Forager**	**Nurse**	**Nurse-age, hive cage**	**Nurse-age, lab cage**
**Onset**	0.952±0.007 (4)	0.869±0.004 (4)	0.756±0.258 (2)	0.850±0.018 (4)
**Median**	0.929±0.012 (4)	0.895±0.028 (4)	0.775±0.194 (2)	0.896±0.016 (4)
**Offset**	0.815±0.053 (4)	0.804±0.063 (4)	0.608±0.091 (2)	0.779±0.058 (4)
